# Detection of Candidate Circular RNAs to Monitor Anti-Hormonal Response in the Mammary Gland

**DOI:** 10.64898/2026.03.26.714379

**Published:** 2026-03-30

**Authors:** Nico Trummer, Malte Weyrich, Paige Ryan, Priscilla A. Furth, Markus Hoffmann, Markus List

**Affiliations:** 1 Data Science in Systems Biology, School of Life Sciences, Technical University of Munich, Freising, Germany; 2 Department of Biochemistry and Molecular & Cellular Biology, Georgetown University Medical Center, Washington, DC, USA; 3 Georgetown University, Washington, DC, USA

## Abstract

Anti-hormonal therapies such as selective estrogen receptor modulators like tamoxifen or aromatase inhibitors like letrozole represent a cornerstone for breast cancer prevention and therapy of estrogen receptor-positive breast cancer. Therapeutic monitoring can include blood tests and imaging; however, genetically-based approaches are not yet in practice. Ideally, a test would be able to detect a positive molecular response across different estrogen pathway-suppressive approaches. Circular RNAs are a species of non-coding RNAs detectable in plasma that have been proposed as non-invasive therapeutic biomarkers. To determine whether a set of specific circular RNAs is altered across estrogen-suppressive pathway approaches, we analyzed mammary gland-specific total RNA sequencing data from two individual genetically engineered mouse models (GEMMs) of estrogen pathway-induced breast cancer, with or without exposure to tamoxifen or letrozole. The nf-core/circrna pipeline was used to identify circRNAs that were differentially expressed in response to either tamoxifen or letrozole. We then screened for circRNAs that were differentially regulated by both anti-hormonals. Four up-regulated and 31 down-regulated circRNAs with host genes known to be expressed in human breast epithelial cells were identified as showing reproducible differential regulation in response to anti-hormonal treatment.

## Introduction

Circular RNAs (circRNAs) represent a species of generally non-coding RNAs that have the capacity to serve as biomarkers, therapeutic targets and to influence downstream gene expression networks (1, 2). CircRNAs are primarily classified as long non-coding RNAs (lncRNAs), although a few of them encode proteins (3). They are formed through a back-splicing event of precursor messenger RNA (pre-mRNA), resulting in the fusion of 5’ and 3’ ends of a portion of pre-mRNA that is spliced out (4). Their closed circular loop structure makes them less prone to degradation (5). circRNAs can be transported to the extracellular space in exosomes (6, 7). Eventually, tissue-generated circRNAs that are secreted in exosomes can be found circulating in the blood and are considered possible biomarkers of response that have the benefit of being assessed using liquid biopsy (8). CircRNAs are generally expressed at levels between two and 10% of that of their linearly expressed host genes, and it is possible for a single host gene to use alternative back-splicing mechanisms to produce multiple circRNA isoforms (6, 7). In some circumstances, multiple circRNAs share the same back-splice junction (9). For use as biomarkers, previous studies have documented that a minimum of a statistically significant 2-fold change (log2fold change of one) is recommended, with higher fold changes proving more reliable, both up- and down-regulated biomarkers are useful, and the use of three or more individual circRNA biomarkers in combination provides a more robust analysis (10, 11)

Anti-hormonal selective estrogen modulators (SERMs) such as tamoxifen and aromatase inhibitors (AIs) such as letrozole represent two of the major therapeutic classes of drugs used for the reduction of breast cancer risk and/or therapy of estrogen receptor-positive breast cancer (12). Here, we investigated the hypothesis that there would be common circRNAs that would be differentially regulated by both classes of antihormonals, which would represent candidate circRNAs that could be monitored for a positive response to anti-hormonal therapy. The nf-core/circrna detection pipeline (13, 14) was used to identify genetic regions containing BSJs representing candidate circRNAs that were differentially regulated following anti-hormonal exposure. The datasets analyzed were total RNA sequencing data of mammary gland tissue derived from a time-course experiment that employed two genetically engineered mouse models (GEMM) of estrogen pathway-induced breast cancer that exhibited the expected loss of mammary gland cellularity and decrease in preneoplasia and neoplasia following exposure to either tamoxifen or letrozole (15). Previous published work has concentrated on identifying circRNAs whose expression is associated with tamoxifen resistance (16–19), and, in the case of AIs, circRNAs that are predictive of a durable AI response (20). Here, we extend our understanding of the relationship between circRNAs and anti-hormonals by identifying candidate circRNAs that are differentially regulated in the context of a positive therapeutic response in whole mammary tissue. Mammary gland tissue from GEMM have been shown to be informative preclinical molecular models of genetic response to therapeutic interventions for human breast cancer prevention and treatment (21, 22). CircRNAs can have orthologous structures in different species, such as mice and humans. These orthologous circRNAs may exhibit conserved genomic origins or have emerged convergently, with variation in conserved function and expression (23, 24).

Here, we used a bioinformatic approach (see Materials and Methods for details) to identify four upregulated and 31 downregulated regions containing BSJs representing candidate circRNAs commonly differentially regulated by both tamoxifen and letrozole in the mouse mammary gland. Two of the up-regulated and 24 of the down-regulated candidate circRNAs have analogous regions in humans containing previously identified circRNAs, representing a first set of potential circRNA biomarkers that could be used to assess anti-hormonal response in human breast tissue at risk for breast cancer development. Five additional candidate circRNA regions identified (one up-regulated and four down-regulated) that have host genes known to be expressed in human breast cells but are not currently listed in any human circRNA database may represent newly identified circRNA structures responsive to antihormonal therapy.

## Results

### Detection of differentially expressed candidate circRNA regions in mouse mammary gland following exposure to tamoxifen or letrozole

**Figure 1.**
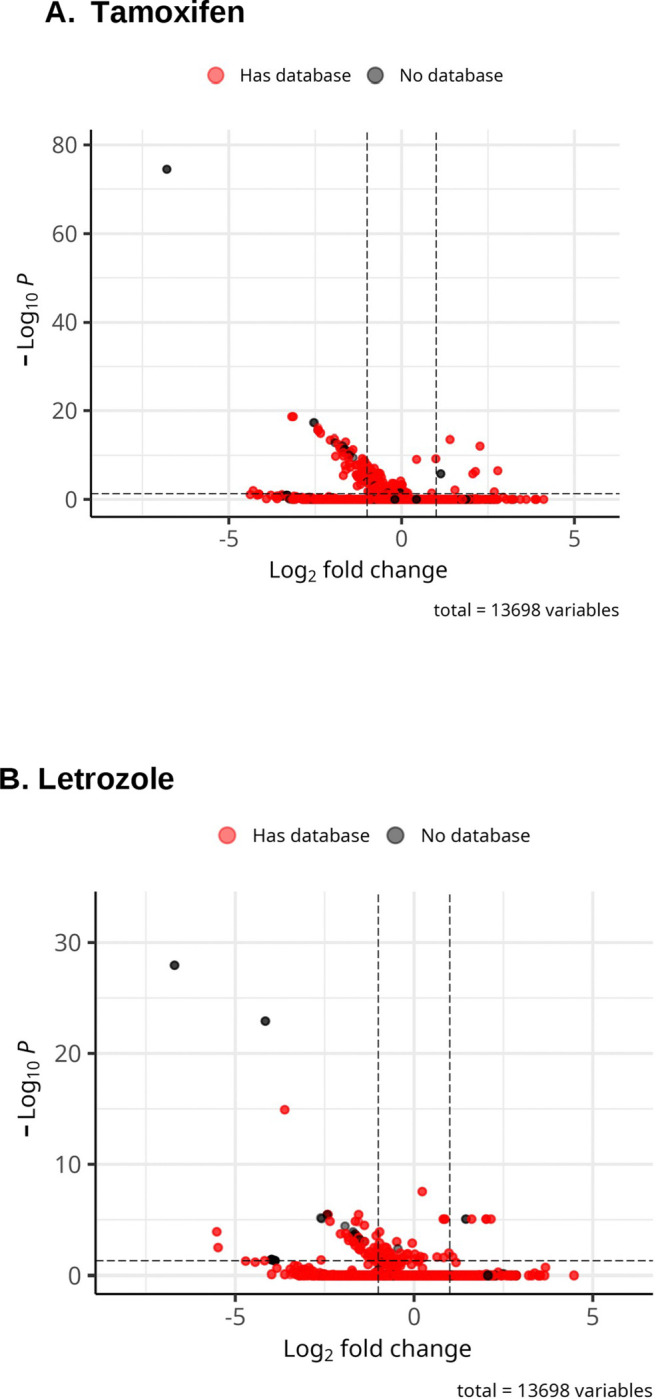
Volcano plots of significantly expressed circRNAs after pre-filtering and lfc shrinkage for both letrozole and tamoxifen. **A.** Volcano plot of significantly expressed circRNAs following tamoxifen exposure. **B.** Volcano plot of significantly expressed circRNAs following letrozole exposure. Any circRNA having a padj < 0.05 and abs(lfc) > 1 was considered significant. CircRNAs that are highlighted red indicate there is an annotation in either circAtlas (53) circBase (52), or the lifted circBase database as described in the methods section.

**Figure 2.**
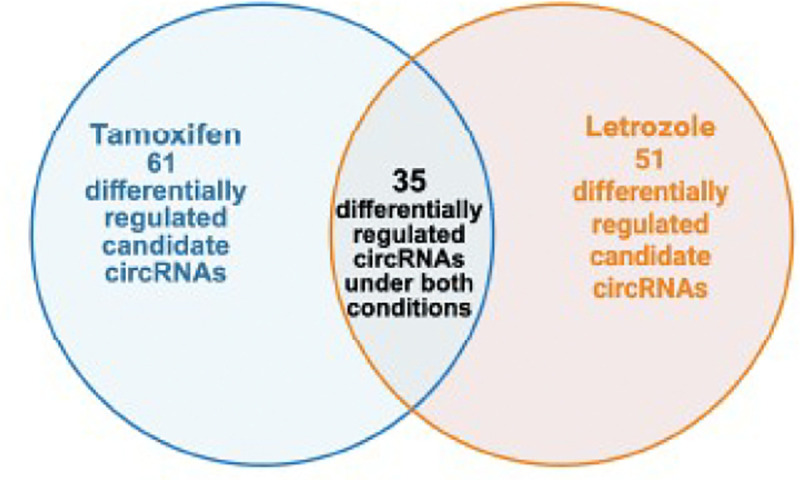
Identification of candidate circRNAs significantly differentially expressed with both tamoxifen and letrozole exposure. Venn diagram of the overlap of differentially expressed circRNAs with tamoxifen versus letrozole exposure. Twenty-one differentially expressed candidate circRNAs were identified using the nf-core/circRNA pipeline and Dseq2 to identify differentially expressed circRNAs under different hormonal conditions (padj. <0.05). Created in BioRender: Furth, P. (2026) https://BioRender.com/x0xbrcl

For the detection of candidate tamoxifen-regulated circRNAs, total RNAseq data from mammary glands of both mammary tumor virus–reverse tetracycline–controlled transactivator (*MMTV-rtTA*)/Tet-operator (*tet-op*)–*Esr1* and mouse mammary tumor virus–reverse tetracycline–controlled transactivator/*tet-op*–*CYP19A1* mice with and without tamoxifen exposure were compared. For the detection of candidate letrozole regulated circRNAs, total RNAseq data from mammary glands of both *MMTV-rtTA/tet-op-Esr1* and *MMTV-rtTA/tet-op-CYP19A1* with and without letrozole exposure were compared. The two different GEMMs analyzed were chosen to represent two major, distinctly different molecular pathways underlying estrogen pathway-related human breast cancer generation, Estrogen Receptor alpha over-expression as compared to aromatase over-expression that increases mammary gland localized estrogen levels (15, 25–27). Application of the nf-core/circrna detection pipeline in combination with DESeq2 resulted in the identification of 61 candidate circRNA regions differentially regulated by tamoxifen exposure and 51 candidate circRNAs differentially regulated by letrozole exposure (adj. p≤0.05) (Figure 1). Data were examined to determine how many of these candidate circRNAs were differentially expressed in the same direction (either up-regulated or down-regulated) by both tamoxifen and letrozole. Thirty-five circRNAs commonly differentially regulated by both tamoxifen and letrozole were identified (Figure 2). Chromosomal coordinates of the candidate circRNA regions, names of candidate host genes within the chromosomal coordinates were determined, and annotation to murine circRNA databases (CircAtlas, CircBase, CircBank, Circpedia, Tissue-Specific CircRNA Database (TSCD) was performed (Tables 1–4). Overall, 33 of the 35 detected circRNAs (94%) in mouse mammary tissue were supported by entries in at least one of the murine circRNA databases, indicating that the pipeline was able to detect true circRNAs as defined by their record in established databases.

### Characterization of candidate circRNA regions commonly differentially regulated by exposure to tamoxifen and letrozole.

Four candidate circRNA regions were found to be significantly up-regulated and 31 candidate circRNA regions were found to be significantly down-regulated and by exposure to both tamoxifen and letrozole (padj<0.05) (Tables 1, 2). Predicted host genes derived from the chromosomal coordinates of the candidate circRNA regions, basemean (average normalized count of the candidate region across all samples), log2foldchange (measure of how much a value has changed between two conditions expressed on a logarithmic scale), lfcSE (standard error estimate for the log2 fold change estimate), stat (log2FoldChange divided by lfcSE, which is compared to a standard normal distribution to generate a two-tailed pvalue), pvalue (probability of observing the measured difference in gene expression between groups assuming the null hypothesis, uses the Wald test), and padj (adjusted p-value) were determined. Average normalized count values (base mean) ranged from 0.46 to 1.58 for up-regulated and 1.03 to 214.05 for down-regulated candidate circRNAs with statistically significant log2fold changes ranging from 1.13 to 2.78 for up-regulated and −1.02 to −6.79 for down-regulated candidate circRNAs (Tables 1, 2). Two candidate down-regulated circRNAs mapping to a transcript set (Rn18s-rs5, Gm26917, AY036118) were marked individually by the pipeline but differed by only two nucleotides at the start and therefore appeared to represent the same general circRNA structure, leaving a total of 30 individual candidate down-regulated circRNAs. In two cases, the candidate circRNA regions mapped to two adjacent genes. One was an up-regulated region on chromosome 7 mapped to *Gm34744* (a predicted long non-coding (lnc) RNA in mice) and *Bcam. Gm34744* is. One down-regulated region on chromosome 6 mapped to *Gm52873* (predicted mouse gene) and *Washc2*. A relative comparison of baseline host gene in transcripts per million (TPM) and basemean circRNA expression levels showed Circular-to-Linear Ratios (CLR) ranging from 0.002 to 0.60 (Supplementary Tables 1, 2). All of the identified circRNAs revealed a fold-change of greater than 2-fold, and 4-fold changes were documented for 20 of the 31 down-regulated circRNAs (Table 2), meaning that the collection of circRNAs identified included multiple candidates that could be used for a combination circRNA biomarker platform. Host gene expression was examined to determine if the differentially regulated circRNA expression identified might be secondary to significant changes in host gene expression following exposure to tamoxifen or letrozole. No instance of host gene expression values paralleling circRNA expression levels across both anti-hormonal exposures and both models was identified although it was noted that for the up-regulated circRNA from host gene *Bcam,* mammary glands from *MMTV-rtTA/tet-op-Esr1* mice showed significantly increased host gene expression with both tamoxifen and letrozole, although the changes were less than 2-fold (Supplementary Table 1). Similarly, the down-regulated circRNAs mapped to host genes *Rpn1* and *Strbp* showed significant decreases with both tamoxifen and letrozole in the mammary glands from *MMTV-rtTA/tet-op-Esr1* mice, but the changes were less than 2-fold, while in all cases the changes in the circRNA expression levels were greater than 2-fold (Supplementary Table 2). Finally, the host gene set belonging to the circRNAs identified was then assessed through Gene Set Enrichment Analysis (GSEA) through both mouse and human gene sets to determine if the Molecular Signatures Database (MSigDB) would identify the set as having any statistically significant relationships to established gene sets. Statistically significant relationships to C3 (28) regulatory target gene sets, C2 curated gene sets, and M3 regulatory target gene sets were identified (data not shown) but not for any hallmark gene sets particularly either HALLMARK_ESTROGEN_RESPONSE_EARLY(M5907) or HALLMARK_ESTROGEN_RESPONSE_LATE(M5906) gene sets. It is known that circRNAs can show differential expression independent of host gene expression patterns (29, 30). Not only are the majority (94%) of identified differentially regulated circRNAs in one of the established mouse circRNA databases but those that are listed are all present in at least two of the queried databases and 12 of the 33 identified circRNAs mapped to more than one listing in at least one of the databases examined (Tables 3, 4). To determine which of the candidate circRNAs identified in mouse mammary gland tissue were plausible candidate circRNA biomarkers for human breast tissue, host gene expression in human breast tissue was examined and identification of orthologous human circRNAs in human circRNA databases was performed. Host genes for three of the four up-regulated and 27 of the 30 down-regulated circRNAs (overall 88%) were documented to be expressed in human breast primary epithelial cells (Tables 5, 6). Human orthologous circRNAs were identified for 25 of the 30 host genes expressed in human breast. Five of the orthologous circRNAs have been previously documented as being expressed in human breast (n=4 in CircAtlas: hsa-FMN1_0002, hsa-CSNK1G3_0001, hsa-ZNF148_0018, hsa-MBNL1 and n=1 in TCSD: circRpn1).

## Discussion

Using a bioinformatic approach, this study demonstrated that there is a set of circRNAs expressed in mammary tissue that undergo statistically significant changes in expression coincident with a positive therapeutic response to antihormonals. Development of a circRNA panel that could serve as biomarkers for a positive therapeutic response could help identify early treatment failures for current agents and assist in validating new anti-hormonals currently under development (31). Currently, clinical strategies for assessing an early therapeutic response to anti-hormonals rely on measuring estrogen levels following aromatase inhibitor treatment to ensure they drop (32) or determining if breast density decreases with anti-hormonal exposure (33, 34). Because changes in circRNA expression can be measured in circulating blood (35–37), they represent a non-invasive means of assessing the molecular response to anti-hormonals.

Preclinical development programs in oncology commonly exploit translational models for initial identification and characterization of circRNA biomarkers (38–40). Here, two GEMMs were used to increase generalizability of the bioinformatic analyses by validating if the same circRNAs were differentially expressed across two different mechanisms of human breast cancer generation and two different classes of anti-hormonal agents. The design of the bioinformatic pipeline for identification of candidate circRNAs was optimized for rigor and reproducibility by focusing only on circRNAs found to be present in all biological samples tested and filtering for circRNAs that exhibited at least a 2-fold difference in expression levels. Current recommendations suggest that any biomarker test utilizing circRNAs include at least three distinct circRNAs (10, 11, 41). Here, we identified 25 distinct candidate orthologous circRNAs that are derived from host genes expressed in human breast, a reasonable collection to begin paring down to find at least three circRNAs that might exhibit measurable changes in expression in human peripheral blood. These experimental results show a proof of principle supporting direct studies in human tissue to discover even more circRNAs relevant to hormone response in both breast and other reproductive tissues, including the possibility of using circRNAs as therapeutic agents (37).

Limitations of the study include the relatively small number of each genotype/condition set of samples (n=3). In this study the small sample size was mitigated by combining together both GEMM for identification of statistically significant changes in circRNA expressions, increasing the sample size for each comparison to n=6. The study was designed as a bioinformatic analysis to establish proof of principle. Future studies will be needed to validate expression changes directly in mouse and human tissue. In addition, while this study focused on differential circRNA abundance, we did not test whether the identified candidates engage in miRNA sponging or other miRNA-linked mechanisms, which will require dedicated computational prediction and experimental validation (42–44). For a circRNA to be an optimal biomarker, it should be secreted in an exosome so that it may be trafficked to the peripheral blood. The candidate circRNAs identified in this study have not yet been assessed for their exosome secretion patterns. Together, these results position circRNAs as a promising, mechanism-agnostic molecular readout of effective estrogen-pathway suppression, establishing a rational foundation for future translational validation of a minimally invasive biomarker panel.

## Methods

### Mouse Models and Total RNA Sequencing Data

Total single-ended RNA sequencing data analyzed in this project was downloaded from the National Center for Biotechnology Information's Gene Expression Omnibus (GSE70440, GSE201767). Total RNA sequencing fastq files were obtained from mammary tissue obtained from cohorts of mammary tumor virus–reverse tetracycline–controlled transactivator/Tet-operator (*tet-op*)–*Esr1* and mouse mammary tumor virus–reverse tetracycline–controlled transactivator/*tet-op*–*CYP19A1* mice on a C57Bl/6 background from experiments examining the impact of age, *Esr1* or *CYP19A1* over-expression, or exposure to tamoxifen or letrozole (n=3 independent mammary gland samples per condition) (15, 25). Studies on the mice were approved by the Georgetown University Animal Care and Use Committee (Protocol number 2018–0022, Progression and regression of neoplasia in aged mice, protocol approval date: 05/01/2018).

### Identification of circRNAs differentially regulated by exposure to either tamoxifen or letrozole using the nf-core/circRNA pipeline.

To identify circRNAs differentially regulated by tamoxifen exposure in both models, an nf-core/circrna detection pipeline (13, 14) was used on the total single-ended RNA sequencing data from mammary glands of tet-op-Esr1 and tet-op-CYP19A1 mouse models (age 20 months) with and without a two-month exposure to tamoxifen, combined data from the mammary glands of tet-op-Esr1 and tet-op-CYP19A1 mouse models (age 20 months) with and without a two-month exposure to letrozole, combined data from mammary glands of tet-op-Esr1 mice with and without induction of the tet-op-Esr1 transgene using GRCm39 as the reference genome (https://www.ncbi.nlm.nih.gov/datasets/genome/GCF_000001635.27/, accessed 11/20/2025). Sequencing was performed using the Illumina NextSeq 550, SE 75-bp read length; minimum reads 50 million per sample. The nf-core/circrna detection pipeline utilizes different tools for back-splice junction (BSJ) detection (CIRIquant (45), CircExplorer2 (46), CircRNA finder (47), DCC (48), Find circ (49), MapSplice (50), Segemehl (51)), annotates the detected circRNAs using Gene Transfer Format (GTF)-based and database-based annotation and extracts the sequences of the circRNAs and quantifies their expression. For database annotation, circBase (52) and circAtlas (53) were used. circBase was lifted over from its original reference to the mm39 reference genome using the University of California Santa Cruz (UCSC) liftOver tool (54). The length of the overlap (latest start and the first end of the identified differentially expressed circRNA and the database entry) has to be at least 90% of the length to be considered a match.

### Differential expression analysis and identification of reproducibly differentially expressed circRNAs under different hormonal conditions.

DESeq2 (55) was used to identify differentially expressed circRNAs (56–58) in two analyses. The pipeline detected 56,621 unique circRNAs (defined by chr:start–stop:strand coordinates). Due to the sparsity of the raw count data, circRNAs were pre-filtered to retain only those with at least 10 counts in at least three samples, resulting in 13,803 candidates for differential expression testing. For both analyses, the design formula "~ age + transgene + induction + drug" was used to control for potential confounding effects of age, transgene status, and induction while assessing drug-associated expression changes. Differential expression was evaluated using contrasts comparing treated versus untreated samples ("contrast = c("drug", "tamoxifen", "no")" and "contrast = c("drug", "letrozole", "no")"). Log2 fold change shrinkage (55) was applied to account for the high variance in effect size estimation, and circRNAs with an absolute log2 fold change greater than 1 were considered for interpretation. P-values were adjusted for multiple testing using the Benjamini–Hochberg method (59), and circRNAs with an adjusted p-value (padj) < 0.05 were considered statistically significant. To find circRNAs that were reproducibly differentially regulated after both tamoxifen or letrozole exposure, circRNAs that were significantly differentially regulated in the same direction with both tamoxifen and letrozole exposure were identified. Chromosomal coordinates had to be within 1 basepair to be considered the same circRNA, and detection of the BSJ had to be found in both + and – amplified cDNA strands of the RNAseq.

### Database Annotation

Initial database annotation was performed as part of the nf-core/circrna detection pipeline. Subsequently, a secondary database annotation was performed on all candidate circRNAs identified. These circRNA entries were extracted and stored in BED format. Databases used for the secondary annotation were circAtlas 3.0 (https://ngdc.cncb.ac.cn/circatlas/, accessed 23 November 2025) (53), CircBase (https://www.circbase.org/, accessed 23 November 2025 (52); Circbank (https://www.circbank.cn/#/home, accessed 23 November 2025) (60), CIRCpedia v3 (https://bits.fudan.edu.cn/circpediav3/, accessed 23 November 2025) (61), and Tissue-Specific CircRNA Database (TSCD) (http://gb.whu.edu.cn/TSCD/, accessed 23 November 2025) (62). To conduct the secondary annotation, all available mouse and human circRNA annotations were downloaded from the circAtlas 3.0 (53), CircBase (52), CIRCpedia v3 (61), and TSCD databases. The downloaded database files were then converted into a standard BED format for subsequent analyses. Genomic coordinates from the database files were mapped to the target assembly using the UCSC LiftOver tool, which converts genomic coordinates between assemblies and identifies corresponding aligned regions (54). Human coordinates from the hg19 and hg38 genome assemblies were lifted to the mm39 mouse genome assembly, and mouse entries were lifted from mm9 and mm10 to mm39, ensuring all comparisons used the most current mouse reference genome. For cross-species liftovers from human to mouse, the minMatch LiftOver parameter was reduced to 0.1 to maximize retrieval of putative orthologous regions. All default parameters were used for mouse genome conversions. It should be noted that the LiftOver tool identifies approximate homologous genomic regions, but does not assess sequence similarity or functional equivalence. Consequently, the resulting mappings do not account for sequence conservation, nor do they imply functional orthology. Once all of the database files were converted to a compatible reference assembly, overlaps between the candidate circRNA data and database annotations were assessed using bedtools intersect (63). Bedtools intersect identifies overlapping genomic features and provides flexible parameters for defining and reporting these overlaps. The parameters -f 0.9 -r -loj -wa -wb were used, ensuring that circRNAs were only considered a match if their genomic coordinates overlapped by at least 90%. The same parameters were applied to all human and mouse datasets.

### Characterization of host gene expression in mouse mammary gland and human breast.

To evaluate host gene expression levels for the identified circRNAs, RNAseq data was analyzed to determine transcripts per million (TPM) values in mammary glands of 20-month-old female mice without exposure to tamoxifen or letrozole (n=3 each genotype) for each host gene (GSE70440) (25). Mean TPM values for each genotype/condition were calculated using https://www.calculatorsoup.com/calculators/statistics/mean-median-mode.php. In an adaptation of a previously published method (64), relative circular over linear ratios (CLR) were calculated (circular basemean/linear TPM) using the basemean levels calculated by the pipeline for the coordinates of the identified circRNAs over transcripts per million (TPM) of the linear forms, a value that corrected for both sequencing depth (total read count) and gene length for the linear forms. Evaluation of host gene differential expression following tamoxifen or letrozole exposure was determined using DEseq2 as described previously (n=3 each genotype/condition) (25). For evaluation of host gene expression in human breast tissue, breast tissue specific RNA expression data from The Human Protein Atlas https://www.proteinatlas.org (last accessed February 6, 2026) was recorded (65, 66) and average fragments per kilobase of transcript per million mapped reads (avgFPKM) for n=18 independent samples of primary human high-risk breast cells determined as described previously (67). Gene Set Enrichment Analysis (GSEA) was performed on mouse and human sets of host genes for the circRNAs identified for assessment of statistically significant relationships with known gene sets (hallmark gene sets, C3 regulatory target gene sets, C2 curated gene sets, M3 regulatory target gene sets) https://www.gsea-msigdb.org/gsea/msigdb/index.jsp (accessed February 5, 2026) (28, 68–70).

## Supplementary Material

Supplement 1

## Figures and Tables

**Table 1.** Candidate circRNA regions identified as commonly up-regulated by tamoxifen and letrozole exposure in mouse mammary gland.

	Host Gene(s)	Chromosome coordinates	baseMean	log2Fold Change	lfcSE	stat	pvalue	padj
1	***Fmn1***							
	Tamoxifen	chr2:113256866-113274804:+	0.455037846	2.059145766	1.556773702	5.574537857	2.48E-08	1.62E-06
		chr2:113256866-113274804:-						
	Letrozole	chr2:113256866-113274804:+		2.018148272	1.557025921	5.546192441	2.92E-08	8.77E-06
		chr2:113256866-113274804:-						
2	***Gm34744, Bcam*** ^1^							
	Tamoxifen	chr7:19499259-19499634:+	1.164347023	2.782265638	1.559762694	5.904174008	3.54E-09	3.18E-07
		chr7:19499258-19499634:-						
	Letrozole	chr7:19499259-19499634:+		2.017345992	1.558684947	5.550537659	2.85E-08	8.77E-06
		chr7:19499258-19499634:-						
3	***Hmg20a***							
	Tamoxifen	chr9:56381814-56389923:+	1.581123477	2.135573096	1.560257849	5.822463697	5.80E-09	4.84E-07
		chr9:56381814-56389923:-						
	Letrozole	chr9:56381814-56389923:+		1.611106227	1.559889878	5.557165583	2.74E-08	8.77E-06
		chr9:56381814-56389923:-						
4	***Scn4a***							
	Tamoxifen	chr11:106220822-106236555:+	1.182666062	1.132328441	1.559717347	5.581742143	2.38E-08	1.58E-06
		chr11:106220823-106236555:-						
	Letrozole	chr11:106220822-106236555:+		1.449359383	1.560351705	5.564453362	2.63E-08	8.77E-06
		chr11:106220823-106236555:-						

Chromosomal coordinates shown for both + and - amplified cDNA strands where BSJs detected for the candidate circRNAs independently detected in comparisons of tamoxifen or letrozole as compared to controls without exposure. Basemean (average normalized count of a gene across all samples), log2foldchange (measure of how much a value has changed between two conditions expressed on a logarithmic scale), IfcSE (standard error estimate for the log2 fold change estimate), stat (log2FoldChange divided by IfcSE, which is compared to a standard normal distribution to generate a two-tailed pvalue), pvalue (probability of observing the measured difference in gene expression between groups assuming the null hypothesis, uses the Wald test), padj (adjusted p-value) listed. When chromosomal region covers more than one known or predicted gene, both are listed.

1 Two distinct, adjacent genes located on mouse Chromosome 7. *Gm34744* is a predicted mouse gene (lncRNA gene) (https://www.alliancegenome.org/gene/MGI:5593903, accessed 19 January 2026).

**Table 2.** Candidate circRNA regions identified as commonly down-regulated by tamoxifen and letrozole exposure in mouse mammary gland.

	Host Gene	Chromosome coordinates	baseMean	log2Fold Change	lfcSE	stat	pvalue	padj
1	***Csn1s2a***							
	Tamoxifen	chr5:87925915-87926842:+	214.0466095	−6.794068481	1.438490222	−18.80438357	6.95E-79	3.34E-75
		chr5:87925915-87926842:−						
	Letrozole	chr5:87925915-87926842:+		−6.699236068	1.435825005	−11.84301301	2.34E-32	1.12E-28
		chr5:87925915-87926842:−						
2	***Rn18s-rs5, Gm26917, AY036118*** ^1^							
	Tamoxifen	chr17:40158496-40158638:+	11.08284811	−2.418640267	1.660683405	−8.940543675	3.87E-19	2.77E-16
		chr17:40158497-40158638:−						
	Letrozole	chr17:40158496-40158638:+		−2.420662257	1.660776777	−5.875493769	4.22E-09	3.44E-06
		chr17:40158497-40158638:−						
3	***Rn18s-rs5, Gm26917, AY036118*** ^1^							
	Tamoxifen	chr17:40158498-40158638:+	11.369926	−2.40766439	1.660874801	−8.935785005	4.04E-19	2.77E-16
		chr17:40158498-40158638:−						
	Letrozole	chr17:40158498-40158638:+		−2.409601407	1.660967983	−5.83506531	5.38E-09	3.44E-06
		chr17:40158498-40158638:−						
4	***Bud23***							
	Tamoxifen	chr5:135085695-135089684:+	4.566529582	−2.346942926	1.661431459	−8.768926314	1.80E-18	1.08E-15
		chr5:135085696-135089684:−						
	Letrozole	chr5:135085695-135089684:+		−2.35143284	1.661546653	−5.436121604	5.45E-08	1.39E-05
		chr5:135085696-135089684:−						
5	***Alg13***							
	Tamoxifen	chrX:143128607-143134372:+	2.992797554	−1.94637613	1.662910839	−8.422864557	3.67E-17	1.96E-14
		chrX:143128608-143134372:−						
	Letrozole	chrX:143128607-143134372:+		−1.912591186	1.663358701	−4.911850323	9.02E-07	0.000176667
		chrX:143128608-143134372:−						
6	***Atf7ip***							
	Tamoxifen	chr6:136540758-136548536:+	2.413852644	−2.065711347	1.662904635	−8.327948764	8.23E-17	3.59E-14
		chr6:136540758-136548536:−						
	Letrozole	chr6:136540758-136548536:+		−2.056986177	1.66328632	−4.891026635	1.00E-06	0.000188227
		chr6:136540758-136548536:−						
7	***Rmnd5a***							
	Tamoxifen	chr6:71390233-71406144:−	2.631677928	−1.620553711	1.649005961	−8.201034551	2.38E-16	9.53E-14
		chr6:71390234-71406144:+						
	Letrozole	chr6:71390233-71406144:−		−1.622010648	1.649390712	−4.791269965	1.66E-06	0.000260683
		chr6:71390234-71406144:+						
8	***Rpn1***							
	Tamoxifen	chr6:88066971-88067277:+	2.436144227	−1.839375692	1.659448263	−8.049303565	8.33E-16	2.96E-13
		chr6:88066971-88067278:−						
	Letrozole	chr6:88066971-88067277:+		−1.839808566	1.66010973	−4.690163919	2.73E-06	0.000379609
		chr6:88066971-88067278:−						
9	***Eefsec***							
	Tamoxifen	chr6:88329427-88335624:−	2.12190258	−1.784598679	1.659949133	−7.746834723	9.42E-15	2.66E-12
		chr6:88329428-88335624:+						
	Letrozole	chr6:88329427-88335624:−		−1.828391575	1.659954946	−4.535206565	5.75E-06	0.000746169
		chr6:88329428-88335624:+						
10	***Zranb1***							
	Tamoxifen	chr7:132549772-132568423:+	2.171941173	−1.408054369	1.645475889	−7.649011705	2.03E-14	5.10E-12
		chr7:132549772-132568423:−						
	Letrozole	chr7:132549772-132568423:+		−1.380716345	1.646038649	−4.47021492	7.81E-06	0.000952049
		chr7:132549772-132568423:−						
11	***Wdr36***							
	Tamoxifen	chr18:32994079-32996315:+	2.392940266	−1.729052218	1.657193343	−7.597576035	3.02E-14	6.73E-12
		chr18:32994079-32996315:−						
	Letrozole	chr18:32994079-32996315:+		−1.691733402	1.6578856	−4.463035117	8.08E-06	0.000957211
		chr18:32994079-32996315:−						
12	***Ttc3***							
	Tamoxifen	chr16:94184770-94186281:−	2.276157511	−1.47262568	1.662916941	−7.477273454	7.59E-14	1.55E-11
		chr16:94184770-94186282:+						
	Letrozole	chr16:94184770-94186281:−		−1.556564389	1.662699122	−4.667669012	3.05E-06	0.000411688
		chr16:94184770-94186282:+						
13	***Smad1***							
	Tamoxifen	chr8:80070282-80080075:−	3.107206785	−1.69187405	1.653186354	−7.421512343	1.16E-13	2.27E-11
		chr8:80070283-80080075:+						
	Letrozole	chr8:80070282-80080075:−		−1.696684422	1.653687096	−4.446726312	8.72E-06	0.001007924
		chr8:80070283-80080075:+						
14	***Strbp***							
	Tamoxifen	chr2:37530862-37537297:+	1.598918642	−1.508436157	1.645966914	−7.242165507	4.42E-13	8.31E-11
		chr2:37530862-37537297:−						
	Letrozole	chr2:37530862-37537297:+		−1.487678965	1.646781451	−4.190166481	2.79E-05	0.002907179
		chr2:37530862-37537297:−						
15	***Csnk1g3***							
	Tamoxifen	chr18:54028603-54039741:+	3.188848824	−1.682535759	1.6626613	−7.153102428	8.48E-13	1.51E-10
		chr18:54028603-54039741:−						
	Letrozole	chr18:54028603-54039741:+		−1.649550342	1.663172524	−4.275930263	1.90E-05	0.002052045
		chr18:54028603-54039741:−						
16	***Gm52873, Washc2*** ^2^							
	Tamoxifen	chr6:116206183-116218583:+	2.224297529	−1.543030416	1.64360781	−7.003961841	2.49E-12	4.05E-10
		chr6:116206184-116218583:−						
	Letrozole	chr6:116206183-116218583:+		−1.544142478	1.643952923	−4.069319297	4.72E-05	0.004308675
		chr6:116206184-116218583:−						
17	***Usp25***							
	Tamoxifen	chr16:76868531-76873409:+	1.547496329	−1.139542989	1.60701124	−6.954547461	3.54E-12	5.56E-10
		chr16:76868531-76873409:−						
	Letrozole	chr16:76868531-76873409:+		−1.116066819	1.607783348	−4.150351434	3.32E-05	0.00325021
		chr16:76868531-76873409:−						
18	***Iqsec1***							
	Tamoxifen	chr6:90666561-90671832:−	1.720272135	−1.083533794	1.580551393	−6.79749831	1.06E-11	1.50E-09
		chr6:90666562-90671832:+						
	Letrozole	chr6:90666561-90671832:−		−1.084237581	1.581006736	−3.894025051	9.86E-05	0.008155292
		chr6:90666562-90671832:+						
19	***Qser1***							
	Tamoxifen	chr2:104608481-104610605:+	2.252472148	−1.176238352	1.634409411	−6.569619602	5.04E-11	6.54E-09
		chr2:104608482-104610605:−						
	Letrozole	chr2:104608481-104610605:+		−1.239363952	1.634165679	−3.916206087	9.00E-05	0.007638075
		chr2:104608482-104610605:−						
20	***Zfp606***							
	Tamoxifen	chr7:12223497-12223971:+	1.942414379	−1.511949426	1.643162371	−6.571526531	4.98E-11	6.54E-09
		chr7:12223497-12223971:−						
	Letrozole	chr7:12223497-12223971:+		−1.511779997	1.64394687	−4.018108999	5.87E-05	0.005260845
		chr7:12223497-12223971:−						
21	***Ncor1***							
	Tamoxifen	chr11:62229684-62236144:−	1.839242355	−1.278839154	1.615203749	−6.449470193	1.12E-10	1.42E-08
		chr11:62229685-62236144:+						
	Letrozole	chr11:62229684-62236144:−		−1.279710273	1.615614145	−3.764088898	0.000167157	0.011969219
		chr11:62229685-62236144:+						
22	***Rangap1***							
	Tamoxifen	chr15:81600767-81606227:+	2.362462662	−1.321790519	1.631944434	−6.410780496	1.45E-10	1.78E-08
		chr15:81600768-81606227:−						
	Letrozole	chr15:81600767-81606227:+		−1.367697385	1.631901202	−3.783642681	0.00015455	0.011585187
		chr15:81600768-81606227:−						
23	***Uggt1***							
	Tamoxifen	chr1:36247023-36260405:+	2.246309071	−1.08552061	1.580868051	−5.996396569	2.02E-09	1.92E-07
		chr1:36247023-36260405:−						
	Letrozole	chr1:36247023-36260405:+		−1.085793506	1.581435054	−3.7683246	0.000164347	0.011946277
		chr1:36247023-36260405:−						
24	***Fnbp1***							
	Tamoxifen	chr2:30986038-30995369:−	1.551181559	−1.016318018	1.573564476	−5.945588855	2.75E-09	2.57E-07
		chr2:30986039-30995369:+						
	Letrozole	chr2:30986038-30995369:−		−1.034721314	1.573890838	−3.709209995	0.000207907	0.01404836
		chr2:30986039-30995369:+						
25	***Taf4***							
	Tamoxifen	chr2:179576821-179581747:−	1.543614172	−1.156208657	1.59848049	−5.909894732	3.42E-09	3.13E-07
		chr2:179576822-179581747:+						
	Letrozole	chr2:179576821-179581747:−		−1.166125074	1.598930222	−3.566697517	0.000361508	0.021953624
		chr2:179576822-179581747:+						
26	***Zfp148***							
	Tamoxifen	chr16:33241690-33288553:+	1.084033602	−1.144032917	1.624317386	−5.889075734	3.88E-09	3.42E-07
		chr16:33241691-33288553:−						
	Letrozole	chr16:33241690-33288553:+		−1.189552032	1.624027508	−3.444587563	0.000571931	0.02872661
		chr16:33241691-33288553:−						
27	***Itpr2***							
	Tamoxifen	chr6:146316819-146328068:+	2.172405275	−1.027408983	1.567659411	−5.824364008	5.73E-09	4.84E-07
		chr6:146316819-146328068:−						
	Letrozole	chr6:146316819-146328068:+		−1.027855259	1.567970776	−3.407572822	0.000655434	0.031602466
		chr6:146316819-146328068:−						
28	***Ptpn13***							
	Tamoxifen	chr5:103649232-103649778:−	1.555892857	−1.042337426	1.571222846	−5.731993786	9.93E-09	7.87E-07
		chr5:103649233-103649778:+						
	Letrozole	chr5:103649232-103649778:−		−1.043033593	1.571697193	−3.377659388	0.000731056	0.033886375
		chr5:103649233-103649778:+						
29	***Chd2***							
	Tamoxifen	chr7:73140226-73169456:−	1.180450973	−1.080398409	1.580841563	−5.727285003	1.02E-08	7.96E-07
		chr7:73140227-73169456:+						
	Letrozole	chr7:73140226-73169456:−		−1.081048623	1.581430384	−3.339863453	0.000838196	0.037773941
		chr7:73140227-73169456:+						
30	***Mbnl1***							
	Tamoxifen	chr3:60503015-60523194:+	1.033475717	−1.035874147	1.59272751	−5.595369953	2.20E-08	1.48E-06
		chr3:60503015-60523194:−						
	Letrozole	chr3:60503015-60523194:+		−1.079422191	1.592434618	−3.39234256	0.000692977	0.032849379
		chr3:60503015-60523194:−						
31	***Gclc***							
	Tamoxifen	chr9:77682360-77686917:+	2.24755095	−1.314503696	1.632734098	−5.500559865	3.79E-08	2.41E-06
		chr9:77682360-77686917:−						
	Letrozole	chr9:77682360-77686917:+		−1.289033462	1.633524819	−3.547285459	0.000389223	0.02289263
		chr9:77682360-77686917:−						

Chromosomal coordinates shown for both + and - amplified cDNA strands where BSJs detected for the candidate circRNAs independently detected in comparisons of tamoxifen or letrozole as compared to controls without exposure. Basemean (average normalized count of a gene across all samples), log2foldchange (measure of how much a value has changed between two conditions expressed on a logarithmic scale), lfcSE (standard error estimate for the log2 fold change estimate), stat (log2FoldChange divided by lfcSE, which is compared to a standard normal distribution to generate a two-tailed pvalue), pvalue (probability of observing the measured difference in gene expression between groups assuming the null hypothesis, uses the Wald test), padj (adjusted p-value) listed. When chromosomal region covers more than one known or predicted gene, both are listed.

1 Mouse gene AY036118 on chromosome 17 represents a transcript set: a transcription factor (71–73) linked to the ETS of Mus musculus ERF (*Erf1*), Gm42418, and Gm26917 (A 45S pre-rRNA transcripts) serving as a precursor for 28, 18, and 5.8S rRNA [46-50].

2 Gm52873: Mouse predicted gene (https://www.ncbi.nlm.nih.gov/gene/115490366).

**Table 3.** Mouse database circRNA identifier listings for candidate differentially expressed circRNAs up-regulated by both tamoxifen and letrozole in mouse mammary gland.

	Host Gene(s) in region of Identified circRNA	Circ-Atlas	Circ-Base	Circ-bank	CIRC-pedia	TSCD
1	***Fmn1***	mmu-Fmn1_0001, mmu-Fmn1_0006, mmu-Fmn1_0040		mmu_Fmn1_0032000, mmu_Fmn1_0034000, mmu_Fmn1_0044000		
2	***Gm34744, Bcam***	mmu-Bcam_0001		mmu_Bcam_0009000	CIRCMMU_Bcam_3	heart_14wks_SRR1772419
3	***Hmg20a***	mmu-Hmg20a_0007		mmu_Hmg20a_0006000	CIRCMMU_Hmg20a_1	
4	***Scn4a***					

**Table 4.** Mouse database circRNA identifier listings for candidate differentially expressed circRNAs down-regulated by both tamoxifen and letrozole in mouse mammary gland.

	Host Gene(s) in region of Identified circRNA	Circ-Atlas	Circ-Base	Circ-bank	CIRC-pedia	TSCD
1	***Csn1s2a***					
2	***Rn18s-rs5, Gm26917, AY036118***	mmu-Yam1_0009		mmu_AY036118_0007000		
3	***Bud23***	mmu-Wbscr22_0002		mmu_Bud23_0002000	CIRCMMU_Bud23_1	testis_14wks_SRR1772420
4	***Alg13***	mmu-Alg13_0005	mm9_circ_013694	mmu_Alg13_0025000	CIRCMMU_Alg13_33, CIRCMMU_Alg13_5	brain_14wks_2_SRR1772416
5	***Atf7ip***	Atf7ip_0014, Atf7ip_0035		mmu_Atf7ip_0032000, mmu_Atf7ip_0035000	CIRCMMU_Atf7ip_8	
6	***Rmnd5a***	mmu-Rmnd5a_0001, mmu-Rmnd5a_0024		mmu_Rmnd5a_0021000, mmu_Rmnd5a_0023000	CIRCMMU_Rmnd5a_3	
7	***Rpn1***	mmu-Rpn1_0003		mmu_Rpn1_0006000	CIRCMMU_Rpn1_2	
8	***Eefsec***	mmu-Eefsec_0010		mmu_Eefsec_0023000		
9	***Zranb1***	mmu-ENSMUSG00000030849, ENSMUSG00000030967_0008, mmu-ENSMUSG00000030849, ENSMUSG00000030967_0016, mmu-Zranb1_0003	mm9_circ_005606	mmu_Zranb1_0008000, mmu_Zranb1_0013000, mmu_Zranb1_0023000	CIRCMMU_Zranb1_1	brain_14wks_2_SRR1772416
1 0	***Wdr36***	mmu-Wdr36_0003, mmu-Wdr36_0002		mmu_Wdr36_0026000, mmu_Wdr36_0027000	CIRCMMU_Wdr36_5	
1 1	***Ttc3***	mmu-Ttc3_0030		mmu_Ttc3_0005000	CIRCMMU_Ttc3_5	
1 2	***Smad1***	mmu-Smad1_0002		mmu_Smad1_0002000	CIRCMMU_Smad1_1	lung_14wks_SRR1772418
1 3	***Strbp***	mmu-Strbp_0063		mmu_Strbp_0128000	CIRCMMU_Strbp_49	
1 4	***Csnk1g3***	mmu-Csnk1g3_0004	mm9_circ_004424	mmu_Csnk1g3_0006000	CIRCMMU_Csnk1g3_3	
1 5	***Gm52873, Washc2***	mmu-Fam21_0001		mmu_Washc2_0022000	CIRCMMU_Washc2_5	
1 6	***Usp25***	mmu-Usp25_0025		mmu_Usp25_0043000	CIRCMMU_Usp25_14	brain_14wks_2_SRR1772416, brain_20wks_2_SRR1796708
1 7	***Iqsec1***	mmu-Iqsec1_0003	mm9_circ_017841	mmu_Iqsec1_0013000	CIRCMMU_Iqsec1_3	brain_20wks_2_SRR1796708, brain_14wks_2_SRR1772416
1 8	***Qser1***	mmu-Qser1_0001, mmu-Qser1_0024		mmu_Qser1_0030000, mmu_Qser1_0031000	CIRCMMU_Qser1_12	
1 9	***Zfp606***	mmu-Zfp606_0001		mmu_Zfp606_0003000	CIRCMMU_Zfp606_1	
2 0	***Ncor1***	mmu-Ncor1_0061, mmu-Ncor1_0168		mmu_Ncor1_0046000	CIRCMMU_Ncor1_4, CIRCMMU_Ncor1_5	
2 1	***Rangap1***	mmu-Rangap1_0003		mmu_Rangap1_0013000	CIRCMMU_Rangap1_1	brain_14wks_2_SRR1772416
2 2	***Uggt1***	mmu-Uggt1_0019, mmu-Uggt1_0026		mmu_Uggt1_0069000, mmu_Uggt1_0067000	CIRCMMU_Uggt1_9	
2 3	***Fnbp1***	mmu-Fnbp1_0013, mmu-Fnbp1_0031		mmu_Fnbp1_0050000, mmu_chr2_2331000	CIRCMMU_Fnbp1_15	brain_20wks_1_SRR1796707
2 4	***Taf4***	mmu-Taf4a_0001, mmu-Taf4a_0021, mmu-Taf4a_0032		mmu_chr2_1790000, mmu_Taf4_0030000, mmu_Taf4_0032000	CIRCMMU_Taf4_8	brain_14wks_2_SRR1772416, brain_20wks_2_SRR1796708
2 5	***Zfp148***	mmu-Zfp148_0032, mmu-Zfp148_0034, mmu-Zfp148_0010, mmu-Zfp148_0014, mmu-Zfp148_0029	mm9_circ_018765	mmu_Zfp148_0011000, mmu_Zfp148_0014000, mmu_Zfp148_0022000, mmu_Zfp148_0018000, mmu_Zfp148_0026000	CIRCMMU_Zfp148_4	
2 6	***Itpr2***	mmu-Itpr2_0034, mmu-Itpr2_0094		mmu_Itpr2_0123000, mmu_Itpr2_0121000	CIRCMMU_Itpr2_, CIRCMMU_Itpr2_87	thymus_8wks_male_SRR2927743
2 7	***Ptpn13***	mmu-Ptpn13_0002	mm9_circ_012843	mmu_Ptpn13_0010000	CIRCMMU_Ptpn13_1	
2 8	***Chd2***	mmu-Chd2_0029, mmu-Chd2_0070, mmu-Chd2_0170		mmu_Chd2_0120000, mmu_Chd2_0121000, mmu_Chd2_0140000	CIRCMMU_Chd2_25, CIRCMMU_Chd2_27	brain_20wks_1_SRR1796707, brain_14wks_2_SRR1772416
2 9	***Mbnl1***	mmu-Mbnl1_0024		mmu_Mbnl1_0026000	CIRCMMU_Mbnl1_4	heart_14wks_SRR1772419
3 0	***Gclc***	mmu-Gclc_0004		mmu_Gclc_0005000	CIRCMMU_Gclc_1	

**Table 5.** Candidate circRNAs commonly up-regulated by tamoxifen and letrozole exposure are derived from host genes known to be expressed in human breast epithelial cells. Human database circRNA identifier listings presented for orthologous regions.

	Host Gene	Human Host Gene Name	Host Gene		Human circRNA orthologous to identified mouse circRNA				
			Host gene expressed in human breast tissue*	Host gene expression in primary human breast epithelial cells (avgFPKM) (67)	circAtlas [*mean expression (counts per million) shown for human breast if available*]	circBase	Circbank	CIRCpedia	TSCD
**1**	***Fmn1***	Formin 1	yes	1.44	hsa-FMN1_0002 [*0.036*], hsa-FMN1_0024, hsa-FMN1_0037, hsa-FMN1_0071		hsa_FMN1_0012400, hsa_FMN1_0012500, hsa_FMN1_0012600, hsa_FMN1_0012100		
**2**	***Gm34744, Bcam***	Basal cell adhesion molecule (Lutheran blood group)	*Gm34744:* no (mouse specific), *Bcam:* yes	*Gm34744:* no (mouse specific), *Bcam:* 166.24					
**3**	***Hmg20a***	High mobility group 20A	yes	12.29	hsa-HMG20A_0003	hsa_circ_0036428	hsa_HMG20A_0001200	CIRCHSA_HMG20A_11	
**4**	***Scn4a***	Sodium voltage-gated channel alpha subunit 4	yes	Not detected.					

* https://www.proteinatlas.org/. Abbreviations: avgFPKM: (Average Fragments Per Kilobase of transcript per Million mapped reads) for n=18 independent samples of primary human high-risk breast cells)^51^.

**Table 6.** Candidate circRNAs commonly down-regulated by tamoxifen and letrozole exposure are derived from host genes known to be expressed in human breast epithelial cells. Human database circRNA identifier listings presented for orthologous regions.

			Host Gene		Human circRNA orthologous to identified mouse circRNA				
	Host Gene	Human Host Gene Name	Host gene expressed in human breast tissue*	Host gene expression in primary human breast epithelial cells (FPKM) (67)	circAtlas [mean expression (counts per million) shown for human breast if available]	circBase	Circbank	CIRCpedia	TSCD
**1**	***Csn1s2a***	Alpha-S2-casein-like A	no (mouse specific)	no (mouse specific)					
**2**	***Rn18s-rs5, Gm26917, AY036118***		no (mouse specific)	no (mouse specific)					
**3**	***Bud23***	BUD23 RRNA Methyltransferase And Ribosome Maturation Factor	yes	50.41				CIRCHSA_BUD23_8	
**4**	***Alg13***	ALG13 UDP-N-Acetylglucosaminyltransferase Subunit	yes	21.92				CIRCHSA_ALG13_146	
**5**	***Atf7ip***	Activating Transcription Factor 7 Interacting Protein	yes	7.92	hsa-ATF7IP_0023, hsa-ATF7IP_0030		hsa_ATF7IP_0002700, hsa_ATF7IP_0003200	CIRCHSA_ATF7IP_4, CIRCHSA_ATF7IP_23	
**6**	***Rmnd5a***	Required For Meiotic Nuclear Division 5 Homolog A	yes	9.39	hsa-RMND5A_0001, hsa-RMND5A_0006		hsa_RMND5A_0000700, hsa_RMND5A_0000300	CIRCHSA_RMND5A_1, CIRCHSA_RMND5A_13	
**7**	***Rpn1***	Ribophorin I	yes	118.61	hsa-RPN1_0001, hsa-RPN1_0017	hsa_circ_0067230	hsa_RPN1_0003300, hsa_RPN1_0003700	CIRCHSA_RPN1_1	mammarygland_adult_51_year_female
**8**	***Eefsec***	Eukaryotic Elongation Factor, Selenocysteine-TRNA	yes	9.75					
**9**	***Zranb1***	Zinc Finger RANBP2-Type containing 1	yes	4.80	hsa-ZRANB1_0003, hsa-RP11-298, hsa_0002, hsa-ZRANB1_00540	hsa_circ_0020335, hsa_circ_0008915	hsa_ZRANB1_0001400, hsa_ZRANB1_0002200, hsa_ZRANB1_0002500, hsa_ZRANB1_0003900		esophagus_mucosa_adult_54_year_male
**1** **0**	***Wdr36***	WD Repeat Domain 36	yes	6.40				CIRCHSA_WDR36_59	
**1** **1**	***Ttc3***	Tetratricopeptide repeat domain 3	yes	5.87	hsa-TTC3_0002, hsa-TTC3_0166	hsa_circ_0008572	hsa_TTC3_0002000, hsa_TTC3_0004400	CIRCHSA_TTC3_253	
**1** **2**	***Smad1***	SMAD Family Member 1	yes	12.40					
**1** **3**	***Strbp***	Spermatid perinuclear RNA binding protein	yes	7.53	hsa-STRBP_0004, hsa-STRBP_0125, hsa-STRBP_0177		hsa_STRBP_0013700, hsa_STRBP_0014300, hsa_STRBP_0014200	CIRCHSA_STRBP_9	
**1** **4**	***Csnk1g3***	Casein kinase 1 gamma 3	yes	11.84	hsa-CSNK1G3_0001 [1.041], hsa-CSNK1G3_004, hsa-CSNK1G3_0026, hsa-CSNK1G3_0031, hsa-CSNK1G3_0080	hsa_circ_0001522	hsa_CSNK1G3_0001800, hsa_CSNK1G3_0001900, hsa_CSNK1G3_0002100, hsa_CSNK1G3_0003500, hsa_CSNK1G3_0003700	CIRCHSA_CSNK1G3_2	
**1** **5**	***Gm52873, Washc2***	Human equivalents: WASHC2C: WASH Complex Subunit 2C, WASHC2A: WASH Complex Subunit 2A	*Gm52873:* no (mouse specific), WASHC2C: yes. WASHC2A: yes	*Gm52873:* no (mouse specific), WASHC2C: 8.92 WASHC2A: 11.15				CIRCHSA_WASHC2C_52	
**1** **6**	***Usp25***	Ubiquitin Specific Peptidasase 25	yes	12.35				CIRCHSA_USP25_148	
**1** **7**	***Iqsec1***	IQ Motif And Sec7 Domain ArfGEF 1	yes	5.38	hsa-IQSEC1_0001	hsa_circ_0064388	hsa_IQSEC1_0002500	CIRCHSA_IQSEC1_2	
**1** **8**	***Qser1***	Glutamine and Serine Rich 1	yes	5.48					
**1** **9**	***Zfp606***	Zinc Finger Protein 606 (Human: Znf606)	yes	3.60	hsa-ZNF606_0002	hsa_circ_0052320, hsa_circ_0062317	hsa_ZNF606_0001100	CIRCHSA_ZNF74_4, CIRCHSA_ZNF606_2	
**2** **0**	***Ncor1***	Nuclear Receptor Corepressor 1	yes	23.07				CIRCHSA_NCOR1_301	
**2** **1**	***Rangap1***	Ran GTPase Activating Protein 1	yes	65.64				CIRCHSA_RANGAP1_43	
**2** **2**	***Uggt1***	UDP-Glucose Glycoprotein Glucosyltransferase 1	yes	11.84				CIRCHSA_UGGT1_156	
**2** **3**	***Fnbp1***	Formin Binding Protein 1	yes	5.90					
**2** **4**	***Taf4***	TATA-Box Binding Protein Associated Factor 4	yes	3.79	hsa-TAF4_0004, hsa-TAF4_0034		hsa_TAF4_0004000, hsa_TAF4_0004100	CIRCHSA_TAF4_25	adipose_omentum_adult_54_year_male
**2** **5**	***Zfp148***	Zinc Finger Protein 148 (Human: Znf148)	yes	6.27	hsa-ZNF148_0006, hsa-ZNF148_0018 [0.049], hsa-ZNF148_0022, hsa-ZNF148_0048, hsa-ZNF148_0103	hsa_circ_0067098, hsa_circ_0067102	hsa_ZNF148_0004200, hsa_ZNF148_0006100, hsa_ZNF148_0005000, hsa_ZNF148_0006200, hsa_ZNF148_0006300, hsa_DUTP1_0000100, hsa_ZNF148_0006800	CIRCHSA_ZNF148_15, CIRCHSA_ZNF148_23, CIRCHSA_ZNF148_2	
**2** **6**	***Itpr2***	Inositol 1,4,5-Trisphosphate Receptor Type 2	yes	5.29	hsa-ITPR2_0004, hsa-ITPR2_0291, hsa-ITPR2_0252		hsa_ITPR2_0036000, hsa_ITPR2_0036100, hsa_ITPR2_0035800	CIRCHSA_ITPR2_35, CIRCHSA_ITPR2_177	
**2** **7**	***Ptpn13***	Protein Tyrosine Phosphatase Non-Receptor Type 13	yes	10.74	hsa-PTPN13_0001, hsa_circ_0006468		hsa_PTPN13_0006000	CIRCHSA_PTPN13_5	
**2** **9**	***Mbnl1***	Muscleblind Like Splicing Regulator 1	yes	24.59	hsa-MBNL1_0002 [0.037], hsa-MBNL1_0180		hsa_MBNL1_0012500, hsa_MBNL1_0013200	CIRCHSA_MBNL1_26, CIRCHSA_MBNL1_3	
**3** **0**	***Gclc***	Glutamate-Cysteine Ligase Catalytic Subunit	yes	39.88	hsa-GCLC_0002		hsa_GCLC_0007400	CIRCHSA_GCLC_28	esophagus_mucosa_adult_37_year_male

* https://www.proteinatlas.org/. Abbreviations: avgFPKM: (Average Fragments Per Kilobase of transcript per Million mapped reads for n=18 independent samples of primary human high-risk breast cells)^51^.

## References

[1] Wang Z., Deng H., Jin Y., Luo M., Huang J., Wang J., Zhang K., Wang L., Zhou J., Circular RNAs: biology and clinical significance of breast cancer. RNA Biol. 20, 859–874 (2023). 37882644 10.1080/15476286.2023.2272468 PMC10730165

[2] Santer L., Bär C., Thum T., Circular RNAs: A novel class of functional RNA molecules with a therapeutic perspective. Mol. Ther. 27, 1350–1363 (2019). 31324392 10.1016/j.ymthe.2019.07.001 PMC6697450

[3] Zhou W.-Y., Cai Z.-R., Liu J., Wang D.-S., Ju H.-Q., Xu R.-H., Circular RNA: metabolism, functions and interactions with proteins. Mol. Cancer 19, 172 (2020). 33317550 10.1186/s12943-020-01286-3 PMC7734744

[4] Kristensen L. S., Andersen M. S., Stagsted L. V. W., Ebbesen K. K., Hansen T. B., Kjems J., The biogenesis, biology and characterization of circular RNAs. Nat. Rev. Genet. 20, 675–691 (2019). 31395983 10.1038/s41576-019-0158-7

[5] Liu C.-X., Chen L.-L., Circular RNAs: Characterization, cellular roles, and applications. Cell 185, 2016–2034 (2022). 35584701 10.1016/j.cell.2022.04.021

[6] Li X., Yang L., Chen L.-L., The Biogenesis, Functions, and Challenges of Circular RNAs. Mol. Cell 71, 428–442 (2018). 30057200 10.1016/j.molcel.2018.06.034

[7] Pisignano G., Michael D. C., Visal T. H., Pirlog R., Ladomery M., Calin G. A., Going circular: history, present, and future of circRNAs in cancer. Oncogene 42, 2783–2800 (2023). 37587333 10.1038/s41388-023-02780-w PMC10504067

[8] Ma L., Guo H., Zhao Y., Liu Z., Wang C., Bu J., Sun T., Wei J., Liquid biopsy in cancer current: status, challenges and future prospects. Signal Transduct. Target. Ther. 9, 336 (2024). 39617822 10.1038/s41392-024-02021-w PMC11609310

[9] Das A., Rout P. K., Gorospe M., Panda A. C., Rolling circle cDNA synthesis uncovers circular RNA splice variants. Int. J. Mol. Sci. 20, 3988 (2019). 31426285 10.3390/ijms20163988 PMC6721031

[10] Lü L., Sun J., Shi P., Kong W., Xu K., He B., Zhang S., Wang J., Identification of circular RNAs as a promising new class of diagnostic biomarkers for human breast cancer. Oncotarget 8, 44096–44107 (2017). 28484086 10.18632/oncotarget.17307 PMC5546465

[11] Wu J., Li J., Liu H., Yin J., Zhang M., Yu Z., Miao H., Circulating plasma circular RNAs as novel diagnostic biomarkers for congenital heart disease in children. J. Clin. Lab. Anal. 33, e22998 (2019). 31429492 10.1002/jcla.22998 PMC6868410

[12] Drăgănescu M., Carmocan C., Hormone therapy in breast cancer. Chirurgia (Bucur) 112, 413–417 (2017). 28862117 10.21614/chirurgia.112.4.413

[13] Digby B., Finn S. P., Ó Broin P., nf-core/circrna: a portable workflow for the quantification, miRNA target prediction and differential expression analysis of circular RNAs. BMC Bioinformatics 24, 27 (2023). 36694127 10.1186/s12859-022-05125-8 PMC9875403

[14] Hoffmann M., Schwartz L., Ciora O.-A., Trummer N., Willruth L.-L., Jankowski J., Lee H. K., Baumbach J., Furth P. A., Hennighausen L., List M., circRNA-sponging: a pipeline for extensive analysis of circRNA expression and their role in miRNA sponging. Bioinform Adv 3, vbad093 (2023). 37485422 10.1093/bioadv/vbad093 PMC10359604

[15] Furth P. A., Wang W., Kang K., Rooney B. L., Keegan G., Muralidaran V., Zou X., Flaws J. A., Esr1 but Not CYP19A1 Overexpression in Mammary Epithelial Cells during Reproductive Senescence Induces Pregnancy-Like Proliferative Mammary Disease Responsive to Anti-Hormonals. Am. J. Pathol. 193, 84–102 (2023). 36464512 10.1016/j.ajpath.2022.09.007 PMC9768685

[16] Yi J., Du J., Chen X., Nie R.-C., Hu G.-S., Wang L., Zhang Y.-Y., Chen S., Wen X.-S., Luo D.-X., He H., Liu W., A circRNA-mRNA pairing mechanism regulates tumor growth and endocrine therapy resistance in ER-positive breast cancer. Proc. Natl. Acad. Sci. U. S. A. 122, e2420383122 (2025). 40233410 10.1073/pnas.2420383122 PMC11874584

[17] Wu R., Yu S., Bi A., Li Y., Tiek D., Yu K., Xiong H., Shi Q., Mo Z., Yu X., Song X., Yin F., Wang Y., Yi W., Liu M., Li P., Hu B., Le A., Cheng S.-Y., Zhou B., Therapeutic targeting of circTNK2 with nanoparticles restores tamoxifen sensitivity and enhances NK cell-mediated immunity in ER-positive breast cancer. Cancer Lett. 627, 217823 (2025). 40419081 10.1016/j.canlet.2025.217823

[18] Sang Y., Chen B., Song X., Li Y., Liang Y., Han D., Zhang N., Zhang H., Liu Y., Chen T., Li C., Wang L., Zhao W., Yang Q., CircRNA_0025202 regulates tamoxifen sensitivity and tumor progression via regulating the miR-182–5p/FOXO3a axis in breast cancer. Mol. Ther. 27, 1638–1652 (2019). 31153828 10.1016/j.ymthe.2019.05.011 PMC6731174

[19] Hu K., Liu X., Li Y., Li Q., Xu Y., Zeng W., Zhong G., Yu C., Exosomes mediated transfer of circ_UBE2D2 enhances the resistance of breast cancer to tamoxifen by binding to MiR-200a-3p. Med. Sci. Monit. 26 (2020). 10.12659/MSM.922253 PMC7431386 32756532

[20] Smid M., Wilting S. M., Uhr K., Rodríguez-González F. G., de Weerd V., Prager-Van der Smissen W. J. C., van der Vlugt-Daane M., van Galen A., Nik-Zainal S., Butler A., Martin S., Davies H. R., Staaf J., van de Vijver M. J., Richardson A. L., MacGrogan G., Salgado R., van den Eynden G. G. G. M., Purdie C. A., Thompson A. M., Caldas C., Span P. N., Sweep F. C. G. J., Simpson P. T., Lakhani S. R., Van Laere S., Desmedt C., Paradiso A., Eyfjord J., Broeks A., Vincent-Salomon A., Futreal A. P., Knappskog S., King T., Viari A., Børresen-Dale A.-L., Stunnenberg H. G., Stratton M., Foekens J. A., Sieuwerts A. M., Martens J. W. M., The circular RNome of primary breast cancer. Genome Res. 29, 356–366 (2019). 30692147 10.1101/gr.238121.118 PMC6396421

[21] Usary J., Zhao W., Darr D., Roberts P. J., Liu M., Balletta L., Karginova O., Jordan J., Combest A., Bridges A., Prat A., Cheang M. C. U., Herschkowitz J. I., Rosen J. M., Zamboni W., Sharpless N. E., Perou C. M., Predicting drug responsiveness in human cancers using genetically engineered mice. Clin. Cancer Res. 19, 4889–4899 (2013). 23780888 10.1158/1078-0432.CCR-13-0522 PMC3778918

[22] Dabydeen S. A., Furth P. A., Genetically engineered ERα-positive breast cancer mouse models. Endocr. Relat. Cancer 21, R195–208 (2014). 24481326 10.1530/ERC-13-0512 PMC4013173

[23] Jeck W. R., Sharpless N. E., Detecting and characterizing circular RNAs. Nat. Biotechnol. 32, 453–461 (2014). 24811520 10.1038/nbt.2890 PMC4121655

[24] Gruhl F., Janich P., Kaessmann H., Gatfield D., Circular RNA repertoires are associated with evolutionarily young transposable elements. Elife 10 (2021). 10.7554/eLife.67991 PMC8516420 34542406

[25] Furth P. A., Wang W., Kang K., Rooney B. L., Keegan G., Muralidaran V., Wong J., Shearer C., Zou X., Flaws J. A., Overexpression of Estrogen Receptor α in Mammary Glands of Aging Mice Is Associated with a Proliferative Risk Signature and Generation of Estrogen Receptor α-Positive Mammary Adenocarcinomas. Am. J. Pathol. 193, 103–120 (2023). 36464513 10.1016/j.ajpath.2022.09.008 PMC9768686

[26] Frech M. S., Halama E. D., Tilli M. T., Singh B., Gunther E. J., Chodosh L. A., Flaws J. A., Furth P. A., Deregulated estrogen receptor alpha expression in mammary epithelial cells of transgenic mice results in the development of ductal carcinoma in situ. Cancer Res. 65, 681–685 (2005). 15705859 PMC6952528

[27] Díaz-Cruz E. S., Sugimoto Y., Gallicano G. I., Brueggemeier R. W., Furth P. A., Comparison of increased aromatase versus ERα in the generation of mammary hyperplasia and cancer. Cancer Res. 71, 5477–5487 (2011). 21840986 10.1158/0008-5472.CAN-10-4652 PMC3405850

[28] Liberzon A., Subramanian A., Pinchback R., Thorvaldsdóttir H., Tamayo P., Mesirov J. P., Molecular signatures database (MSigDB) 3.0. Bioinformatics 27, 1739–1740 (2011). 21546393 10.1093/bioinformatics/btr260 PMC3106198

[29] Dou Y., Cha D. J., Franklin J. L., Higginbotham J. N., Jeppesen D. K., Weaver A. M., Prasad N., Levy S., Coffey R. J., Patton J. G., Zhang B., Circular RNAs are down-regulated in KRAS mutant colon cancer cells and can be transferred to exosomes. Sci. Rep. 6, 37982 (2016). 27892494 10.1038/srep37982 PMC5125100

[30] You X., Vlatkovic I., Babic A., Will T., Epstein I., Tushev G., Akbalik G., Wang M., Glock C., Quedenau C., Wang X., Hou J., Liu H., Sun W., Sambandan S., Chen T., Schuman E. M., Chen W., Neural circular RNAs are derived from synaptic genes and regulated by development and plasticity. Nat. Neurosci. 18, 603–610 (2015). 25714049 10.1038/nn.3975 PMC4376664

[31] Patel R., Klein P., Tiersten A., Sparano J. A., An emerging generation of endocrine therapies in breast cancer: a clinical perspective. NPJ Breast Cancer 9, 20 (2023). 37019913 10.1038/s41523-023-00523-4 PMC10076370

[32] Dai S., Wu X., Huang X., Li J., Wang X., Wang S., Tang J., Shi Y., Xie X., Xu F., Liu P., Huang J., Xie X., An X., Chen M., Hong R., Xia W., Zheng Q., Jiang K., Zhong Y., Yuan Z., Huang Y., Bi X., Xue C., Clinical significance of serum estradiol monitoring in women receiving adjuvant aromatase inhibitor for hormone receptor-positive early breast cancer. Breast 78, 103818 (2024). 39357125 10.1016/j.breast.2024.103818 PMC11480241

[33] Engmann N. J., Scott C. G., Jensen M. R., Ma L., Brandt K. R., Mahmoudzadeh A. P., Malkov S., Whaley D. H., Hruska C. B., Wu F. F., Winham S. J., Miglioretti D. L., Norman A. D., Heine J. J., Shepherd J., Pankratz V. S., Vachon C. M., Kerlikowske K., Longitudinal changes in volumetric breast density with tamoxifen and aromatase inhibitors. Cancer Epidemiol. Biomarkers Prev. 26, 930–937 (2017). 28148596 10.1158/1055-9965.EPI-16-0882 PMC5457346

[34] Hammarström M., Gabrielson M., Bergqvist J., Lundholm C., Crippa A., Bäcklund M., Wengström Y., Borgquist S., Eliasson E., Eriksson M., Tapia J., Czene K., Hall P., Influence of endoxifen on mammographic density: results from the KARISMA-Tam trial. J. Natl. Cancer Inst. 117, 629–636 (2025). 39514025 10.1093/jnci/djae280 PMC11972671

[35] Zhang J., Luo Z., Zheng Y., Duan M., Qiu Z., Huang C., CircRNA as an Achilles heel of cancer: characterization, biomarker and therapeutic modalities. J. Transl. Med. 22, 752 (2024). 39127679 10.1186/s12967-024-05562-4 PMC11316389

[36] Zhong P., Bai L., Hong M., Ouyang J., Wang R., Zhang X., Chen P., A comprehensive review on circulating cfRNA in plasma: Implications for disease diagnosis and beyond. Diagnostics (Basel) 14, 1045 (2024). 38786343 10.3390/diagnostics14101045 PMC11119755

[37] Wu D., Lu H., Wu J. W. L., Liu B L., Yi X., Wang J., Hailin Tang Amplified electrochemical detection of circular RNA in breast cancer patients using ferrocene-capped gold nanoparticle/streptavidin conjugates. Microchemical Journal 164 (2021).

[38] Liu W., Pan Y., Zhu H., Zhou Y., Zhang H., Liu L., Liu Q., Ji G., CircRNA_0008194 functions as a ceRNA to promote invasion of hepatocellular carcinoma via inhibiting miR-190a/AHNAK signaling pathway. J. Clin. Lab. Anal. 36, e24286 (2022). 35199873 10.1002/jcla.24286 PMC8993631

[39] Tian L., Xu F., Lu Y., Deng Z., Gao Y., Yang J., Exploring a circulating circRNA and miRNA biomarker panel for early detection of ovarian cancer through multiple omics analysis. Sci. Rep. 15, 25809 (2025). 40670591 10.1038/s41598-025-11641-3 PMC12267751

[40] Kersten K., de Visser K. E., van Miltenburg M. H., Jonkers J., Genetically engineered mouse models in oncology research and cancer medicine. EMBO Mol. Med. 9, 137–153 (2017). 28028012 10.15252/emmm.201606857 PMC5286388

[41] Zuo L., Zhang L., Zu J., Wang Z., Han B., Chen B., Cheng M., Ju M., Li M., Shu G., Yuan M., Jiang W., Chen X., Yan F., Zhang Z., Yao H., Circulating Circular RNAs as biomarkers for the diagnosis and prediction of outcomes in acute ischemic stroke. Stroke 51, 319–323 (2020). 31690252 10.1161/STROKEAHA.119.027348

[42] Boniolo F., Hoffmann M., Roggendorf N., Tercan B., Baumbach J., Castro M. A. A., Robertson A. G., Saur D., List M., spongEffects: ceRNA modules offer patient-specific insights into the miRNA regulatory landscape. Bioinformatics, doi: 10.1093/bioinformatics/btad276 (2023). PMC10220456 37084275

[43] Hoffmann M., Pachl E., Hartung M., Stiegler V., Baumbach J., Schulz M. H., List M., SPONGEdb: a pan-cancer resource for competing endogenous RNA interactions. [Preprint] (2021). 10.1093/narcan/zcaa042. PMC8210024 34316695

[44] List M., Dehghani Amirabad A., Kostka D., Schulz M. H., Large-scale inference of competing endogenous RNA networks with sparse partial correlation. Bioinformatics 35, i596–i604 (2019). 31510670 10.1093/bioinformatics/btz314 PMC6612827

[45] Zhang J., Chen S., Yang J., Zhao F., Accurate quantification of circular RNAs identifies extensive circular isoform switching events. Nat. Commun. 11, 90 (2020). 31900416 10.1038/s41467-019-13840-9 PMC6941955

[46] Zhang X.-O., Dong R., Zhang Y., Zhang J.-L., Luo Z., Zhang J., Chen L.-L., Yang L., Diverse alternative back-splicing and alternative splicing landscape of circular RNAs. Genome Res. 26, 1277–1287 (2016). 27365365 10.1101/gr.202895.115 PMC5052039

[47] Jakub O., Westholm P., Miura S., Olson S., Shenker B., Joseph P., Sanfilippo S. E., Celniker B. R., Graveley E. C., Genome-wide Analysis of Drosophila Circular RNAs Reveals Their Structural and Sequence Properties and Age-Dependent Neural Accumulation Westholm Cell Reports (2014). 10.1016/j.celrep.2014.10.062 PMC4279448 25544350

[48] Cheng J., Metge F., Dieterich C., Specific identification and quantification of circular RNAs from sequencing data. Bioinformatics 32, 1094–1096 (2016). 26556385 10.1093/bioinformatics/btv656

[49] Memczak S., Jens M., Elefsinioti A., Torti F., Krueger J., Rybak A., Maier L., Mackowiak S. D., Gregersen L. H., Munschauer M., Loewer A., Ziebold U., Landthaler M., Kocks C., le Noble F., Rajewsky N., Circular RNAs are a large class of animal RNAs with regulatory potency. Nature 495, 333–338 (2013). 23446348 10.1038/nature11928

[50] Wang K., Singh D., Zeng Z., Coleman S. J., Huang Y., Savich G. L., He X., Mieczkowski P., Grimm S. A., Perou C. M., MacLeod J. N., Chiang D. Y., Prins J. F., Liu J., MapSplice: accurate mapping of RNA-seq reads for splice junction discovery. Nucleic Acids Res. 38, e178 (2010). 20802226 10.1093/nar/gkq622 PMC2952873

[51] Hoffmann S., Otto C., Doose G., Tanzer A., Langenberger D., Christ S., Kunz M., Holdt L., Teupser D., Hackermueller J., Stadler P. F., A multi-split mapping algorithm for circular RNA, splicing, trans-splicing, and fusion detection. Genome Biology 15 (2014). 10.1186/gb-2014-15-2-r34 PMC4056463 24512684

[52] Glažar P., Papavasileiou P., Rajewsky N., circBase: a database for circular RNAs. RNA 20, 1666–1670 (2014). 25234927 10.1261/rna.043687.113 PMC4201819

[53] Wu W., Zhao F., Zhang J., circAtlas 3.0: a gateway to 3 million curated vertebrate circular RNAs based on a standardized nomenclature scheme. Nucleic Acids Res. 52, D52–D60 (2024). 37739414 10.1093/nar/gkad770 PMC10767913

[54] Hinrichs A. S., Karolchik D., Baertsch R., Barber G. P., Bejerano G., Clawson H., The UCSC Genome Browser Database: update. Nucleic Acids Res 34, D590–598 (2006). 16381938 10.1093/nar/gkj144 PMC1347506

[55] Love M. I., Huber W., Anders S., Moderated estimation of fold change and dispersion for RNA-seq data with DESeq2. Genome Biol. 15, 550 (2014). 25516281 10.1186/s13059-014-0550-8 PMC4302049

[56] Hu X., Chen L., Wu S., Xu K., Jiang W., Qin M., Zhang Y., Liu X., Integrative analysis reveals key circular RNA in atrial fibrillation. Front. Genet. 10, 108 (2019). 30838031 10.3389/fgene.2019.00108 PMC6389718

[57] Iparraguirre L., Alberro A., Iñiguez S. G., Muñoz-Culla M., Vergara I., Matheu A., Otaegui D., Blood RNA-Seq profiling reveals a set of circular RNAs differentially expressed in frail individuals. Immun. Ageing 20, 33 (2023). 37434183 10.1186/s12979-023-00356-6 PMC10334614

[58] Hoffmann M., Willruth L.-L., Dietrich A., Lee H. K., Knabl L., Trummer N., Baumbach J., Furth P. A., Hennighausen L., List M., Blood transcriptomics analysis offers insights into variant-specific immune response to SARS-CoV-2. Sci. Rep. 14, 1–11 (2024). 38307916 10.1038/s41598-024-53117-w PMC10837437

[59] Powerful Approach to Multiple Testing”. en. Powerful Approach to Multiple Testing”. en. Journal of the Royal Statistical Society: Series B (Methodological) (1995).

[60] Liu M., Wang Q., Shen J., Yang B. B., Ding X., Circbank: a comprehensive database for circRNA with standard nomenclature. RNA Biol. 16, 899–905 (2019). 31023147 10.1080/15476286.2019.1600395 PMC6546381

[61] Zhai S.-N., Zhang Y.-Y., Chen M.-H., Fu Z.-C., Chen L.-L., Ma X.-K., Yang L., CIRCpedia v3: an interactive database for circular RNA characterization and functional exploration. Nucleic Acids Res., doi: 10.1093/nar/gkaf1039 (2025). PMC12807736 41123017

[62] Xia S., Feng J., Lei L., Hu J., Xia L., Wang J., Xiang Y., Liu L., Zhong S., Han L., He C., Comprehensive characterization of tissue-specific circular RNAs in the human and mouse genomes. Brief. Bioinform. 18, 984–992 (2017). 27543790 10.1093/bib/bbw081

[63] Quinlan A. R., Hall I. M., BEDTools: a flexible suite of utilities for comparing genomic features. Bioinformatics 26, 841–842 (2010). 20110278 10.1093/bioinformatics/btq033 PMC2832824

[64] Nicolet B. P., Engels S., Aglialoro F., van den Akker E., von Lindern M., Wolkers M. C., Circular RNA expression in human hematopoietic cells is widespread and cell-type specific. Nucleic Acids Res. 46, 8168–8180 (2018). 30124921 10.1093/nar/gky721 PMC6144802

[65] Uhlen M., Zhang C., Lee S., Sjöstedt E., Fagerberg L., Bidkhori G., Benfeitas R., Arif M., Liu Z., Edfors F., Sanli K., von Feilitzen K., Oksvold P., Lundberg E., Hober S., Nilsson P., Mattsson J., Schwenk J. M., Brunnström H., Glimelius B., Sjöblom T., Edqvist P.-H., Djureinovic D., Micke P., Lindskog C., Mardinoglu A., Ponten F., A pathology atlas of the human cancer transcriptome. Science 357 (2017). 10.1126/science.aan2507 28818916

[66] Karlsson M., Zhang C., Méar L., Zhong W., Digre A., Katona B., Sjöstedt E., Butler L., Odeberg J., Dusart P., Edfors F., Oksvold P., von Feilitzen K., Zwahlen M., Arif M., Altay O., Li X., Ozcan M., Mardinoglu A., Fagerberg L., Mulder J., Luo Y., Ponten F., Uhlén M., Lindskog C., A single-cell type transcriptomics map of human tissues. Sci. Adv. 7, eabh2169 (2021). 34321199 10.1126/sciadv.abh2169 PMC8318366

[67] Alothman S. J., Kang K., Liu X., Krawczyk E., Azhar R. I., Hu R., Goerlitz D., Kallakury B. V., Furth P. A., Characterization of transcriptome diversity and in vitro behavior of primary human high-risk breast cells. Sci. Rep. 12, 6159 (2022). 35459280 10.1038/s41598-022-10246-4 PMC9033878

[68] Subramanian A., Tamayo P., Mootha V. K., Mukherjee S., Ebert B. L., Gillette M. A., Paulovich A., Pomeroy S. L., Golub T. R., Lander E. S., Mesirov J. P., Gene set enrichment analysis: A knowledge-based approach for interpreting genome-wide expression profiles. Proceedings of the National Academy of Sciences 102, 15545–15550 (2005). 10.1073/pnas.0506580102 PMC1239896 16199517

[69] Castanza A. S., Recla J. M., Eby D., Thorvaldsdóttir H., Bult C. J., Mesirov J. P., Extending support for mouse data in the Molecular Signatures Database (MSigDB). Nat. Methods 20, 1619–1620 (2023). 37704782 10.1038/s41592-023-02014-7 PMC11397807

[70] Liberzon A., Birger C., Thorvaldsdóttir H., Ghandi M., Mesirov J. P., Tamayo P., The Molecular Signatures Database (MSigDB) hallmark gene set collection. Cell Syst. 1, 417–425 (2015). 26771021 10.1016/j.cels.2015.12.004 PMC4707969

[71] Hoffmann M., Vaz T., Chhatrala S., Hennighausen L., Data-driven projections of candidate enhancer-activating SNPs in immune regulation. BMC Genomics 26 (2025). 10.1186/s12864-025-11374-7 PMC11863423 40011812

[72] Hoffmann M., Hennighausen L., Spotlight on amino acid changing mutations in the JAK-STAT pathway: from disease-specific mutation to general mutation databases. Sci. Rep. 15, 1–12 (2025). 39979591 10.1038/s41598-025-90788-5 PMC11842829

[73] Hoffmann M., Trummer N., Schwartz L., Jankowski J., Lee H. K., Willruth L.-L., Lazareva O., Yuan K., Baumgarten N., Schmidt F., Baumbach J., Schulz M. H., Blumenthal D. B., Hennighausen L., List M., TF-Prioritizer: a Java pipeline to prioritize condition-specific transcription factors. Gigascience 12 (2022). 10.1093/gigascience/giad026 PMC10155229 37132521

