# Supplementary material for: Detection of Candidate Circular RNAs to Monitor Anti-Hormonal Response in the Mammary Gland
Source: bioRxiv. 2026 Mar 30:2026.03.26.714379. Preprint. [Version 1] doi: 10.64898/2026.03.26.714379 (PMC13060158; doi:10.64898/2026.03.26.714379)
Supplement: Supplement 1 [file NIHPP2026.03.26.714379v1-supplement-1.pdf]

bioRxiv preprint doi: https://doi.org/10.64898/2026.03.26.714379; this version posted March 30, 2026. The copyright holder for this preprint (which was not certified by peer review) is the author/funder, who has granted bioRxiv a license to display the preprint in perpetuity. It is made available under aCC-BY 4.0 International license.

Supplementary Table 1. Expression patterns of host genes in mouse mammary gland for candidate circRNAs identified as commonly up-regulated by tamoxifen and letrozole exposure.

	**Host Gene**	Baseline Mean TPM	Relative Circular-to-Linear Ratio (CLR)	Significantly differentially regulated with letrozole exposure in *CYP19A1* mice	Significantly differentially regulated with letrozole exposure in *Esr1* mice	Significantly differentially regulated with tamoxifen exposure in *CYP19A1* mice	Significantly differentially regulated with tamoxifen exposure in *Esr1* mice
**1**	***Fmn1***	3.89	0.12	no	Sig. down-regulated (log2fold -0.56)	no	no
**2**	***Gm34744*[1], *Bcam***	*Bcam:* 29.69[2]	0.04[3]	*Bcam*: no	*Bcam*: Sig. up-regulated (log2fold 0.51)	*Bcam*: no	*Bcam*: Sig. up-regulated (log2fold 0.53)
**3**	***Hmg20a***	11.27	0.14	no	no	no	no
**4**	***Scn4a***	28.09[4]	0.04	no	no	Sig. down-regulated (log2fold -0.50)	Sig. up-regulated (log2fold 1.23)

Data analyzed from dataset GSE201326 (*15*). Differentially expressed genes (DEGs) between mice exposed to either letrozole or tamoxifen versus controls identified using DESeq2. Genes were considered statistically significantly differentially expressed when the adjusted P value was <0.05. [1]*Gm34744*: full length gene expression not detected in dataset GSE201326. [2] Expressed statistically significantly higher (log2fold 0.61) in *CYP19A1* (mean 35.57) vs. *Esr1* (mean 23.81) mice. [3] Calculated using only *Bcam* TPM as linear *Gm34744* not identified in the analysis of the RNAseq data for linear forms. [4] Expressed statistically significantly higher (log2fold 1.10) in *CYP19A1* (mean 37.72) vs. *Esr1* (mean 18.46) mice. Abbreviations: *Esr1*: *MMTV-rtTA/Tet-op-Esr1*. *CYP19A1*: *MMTV-rtTA/Tet-op-CYP19A1.* Baseline Mean TPM: Mean TPM (Transcripts per million) in mammary gland tissue from 20-month-old female mice without exposure to tamoxifen or letrozole (n=6 samples).

bioRxiv preprint doi: https://doi.org/10.64898/2026.03.26.714379; this version posted March 30, 2026. The copyright holder for this preprint (which was not certified by peer review) is the author/funder, who has granted bioRxiv a license to display the preprint in perpetuity. It is made available under aCC-BY 4.0 International license.

Supplementary Table 2. Expression patterns of host genes in mouse mammary gland for candidate circRNAs identified as commonly down-regulated by tamoxifen and letrozole exposure.

	**Host Gene**	Baseline Mean TPM	Relative Circular-to-Linear Ratio (CLR)	Significantly differentially regulated with letrozole exposure in *CYP19A1* mice	Significantly differentially regulated with letrozole exposure in *Esr1* mice	Significantly differentially regulated with tamoxifen exposure in *CYP19A1* mice	Significantly differentially regulated with tamoxifen exposure in *Esr1* mice
**1**	***Csn1s2a***	3790.42	0.06	no	Significantly down-regulated (log2fold -9.22)	no	no
**2**	***Rn18s-rs5, Gm26917, AY036118***	1062.89	0.01	no	no	no	no
**3**	***Bud23***	13.93	0.33	no	no	no	no
**4**	***Alg13***	5.02	0.60	no	no	no	no
5	***Atf7ip***	29.04[1]	0.08	no	no	no	no
6	***Rmnd5a***	32.54[2]	0.08	no	Significantly up-regulated (log2fold 0.33)	no	no
7	***Rpn1***	49.78[3]	0.05	no	Significantly down-regulated (log2fold -0.59)	no	Significantly down-regulated (log2fold -0.63)
8	***Eefsec***	4.98	0.43	no	no	no	no
9	***Zranb1***	17.65	0.12	no	no	no	no
10	***Wdr36***	12.39	0.19	no	no	no	no
11	***Ttc3***	30.87	0.07	no	no	no	no
12	***Smad1***	9.03	0.34	no	no	no	no
13	***Strbp***	10.78[4]	0.15	no	Significantly down-regulated (log2fold -0.76)	no	Significantly down-regulated (log2fold -0.55)
14	***Csnk1g3***	6.66	0.48	no	no	no	no
15	***Gm52873, Washc2***[5]	*Washc2:* 25.02	*Washc2:* 0.09	*Washc2:* no	*Washc2:* no	*Washc2:* no	*Washc2:* no
16	***Usp25***	29.90	0.05	no	Significantly up-regulated (log2fold	no	no

bioRxiv preprint doi: https://doi.org/10.64898/2026.03.26.714379; this version posted March 30, 2026. The copyright holder for this preprint (which was not certified by peer review) is the author/funder, who has granted bioRxiv a license to display the preprint in perpetuity. It is made available under aCC-BY 4.0 International license.

					0.35)		
17	***Iqsec1***	19.98[6]	0.09	no	Significantly up-regulated (log2fold 0.71)	no	Significantly up-regulated (log2fold 0.74)
18	***Qser1***	9.66	0.23	no	no	Significantly down-regulated (log2fold -0.43)	no
19	***Zfp606***	6.17	0.31	no	no	no	no
20	***Ncor1***	84.93	0.02	no	no	no	no
21	***Rangap1***	10.82	0.22	no	no	no	no
22	***Uggt1***	24.15	0.09	no	no	no	no
23	***Fnbp1***	17.28	0.09	no	no	no	no
24	***Taf4***	5.44	0.28	no	no	no	no
25	***Zfp148***	16.52	0.06	no	no	no	no
26	***Itpr2***	15.76	0.14	no	no	no	no
27	***Ptpn13***	4.63	0.34	no	no	no	no
28	***Chd2***	41.98	0.002	no	no	no	no
29	***Mbnl1***	126.26	0.008	no	Significantly up-regulated (log2fold 0.51)	no	no
30	***Gclc***	12.10	0.19	no	no	Significantly down-regulated (log2fold -0.72)	no

Data analyzed from dataset GSE201326 [4]. Differentially expressed genes (DEGs) between mice exposed to either letrozole or tamoxifen versus controls identified using DESeq2. Genes were considered statistically significant differentially expressed when the adjusted P value was <0.05. [1] Expressed statistically significantly lower (log2fold -0.50) in *CYP19A1* (mean 22.68) vs. *Esr1* (mean 35.39) mice.[2] Expressed statistically significantly higher (log2fold 0.40) in *CYP19A1* (mean 36.22) vs. *Esr1* (mean 28.85) mice. [3] Expressed statistically significantly lower (log2fold -0.37) in *CYP19A1* (mean 43.06) vs. *Esr1* (mean 56.48) mice. [4] Expressed statistically significantly lower (log2fold -0.41) in *CYP19A1* (mean 8.71) vs. *Esr1* (mean 12.85) mice. [5] Only linear *Washc2* detected in dataset GSE201326. [6] Expressed statistically significantly higher (log2fold 0.77) in *CYP19A1* (mean 42.51) vs. *Esr1* (mean 15.45) mice. Abbreviations: *Esr1*: *MMTV-rtTA/Tet-op-Esr1*. *CYP19A1*: *MMTV-rtTA/Tet-op-CYP19A1.*
